# Prevalence and associated factors of foot self-care practice among diabetic patients in Africa: systematic review and meta-analysis

**DOI:** 10.3389/fendo.2025.1527402

**Published:** 2025-06-04

**Authors:** Yibeltal Assefa Atalay, Bersufekad Wubie Alemie, Kelemu Abebe Gelaw

**Affiliations:** ^1^ School of Public Health, College of Health Science and Medicine, Wolaita Sodo University, Wolaita Sodo, Ethiopia; ^2^ Department of Ophthalmology and Optometry, Hawassa University College of Medicine and Health Science, Hawassa, Ethiopia; ^3^ Department of Midwifery, Menelik II Medical and Health Science College, Addis Ababa, Ethiopia

**Keywords:** Africa, associated factors, diabetic patients, foot-care practices, systematic review

## Abstract

**Introduction:**

Nowadays, diabetes mellitus is a major global health issue with serious social, economic, and developmental impacts. One of its most severe complications is foot problems. Research shows that poor self-care practices in managing diabetic feet are a key factor in ulcer development.

**Objective:**

This study aimed to determine the pooled prevalence of foot self-care practice and associated factors among diabetic patients in Africa.

**Methods:**

We found articles using electronic databases, including PubMed, Cochrane Library, Google Scholar, Web of Science, African Journals Online, and Science Direct. Accordingly, we identified 143 published articles. A total of 31 eligible studies were included in the systematic review. Data extraction used a standardized checklist, and analysis was conducted with STATA 14 statistical software. Various methods were used to assess the presence of publication bias, including funnel plots and the Begg and Egger regression test. A significance level of P ≤0.05 was used to indicate potential publication bias. Heterogeneity between included studies was assessed using the I2 statistics. The random effect model was used to estimate the pooled estimates.

**Results:**

The pooled prevalence of foot self-care practices among diabetic patients in Africa was 46.93% (95%CI (39.44–54.41)). Diabetic foot self-care practices were significantly associated with rural residence (AOR: 2.50, 95% CI (1.65–3.80)), educational level (AOR: 3.00, 95%CI (2.07–4.34)), and knowledge level of diabetes patients (AOR: 3.41, 95%CI (2.22–5.23)).

**Conclusions:**

In conclusion, more than half of diabetic patients had poor diabetes foot self-care practices. Diabetic foot self-care practice was associated with a level of education, rural residence, and the knowledge level of diabetic foot care. Therefore, intervention programs ought to prioritize enhancing the knowledge base of individuals with diabetes to promote better self-care practices for their feet.

## 1 Introduction

Diabetes mellitus (DM) is complex group of metabolic disorders that primarily manifest through chronic hyperglycemia or elevated blood glucose levels. This condition arises from defects in insulin secretion, insulin action, or both, leading to a variety of physiological complications. The two common forms of diabetes are type one diabetes, which is characterized by an autoimmune destruction of insulin-producing beta cells in the pancreas, and Type 2 diabetes, which typically involves insulin resistance and a relative deficiency in insulin production (1).

In addition to the health implications, foot ulcers also impose a substantial financial burden on both patients and healthcare systems. Costs associated with treating foot-related problems can be considerable, encompassing expenses related to medical consultations, diagnostic tests, wound care supplies, and potential surgical interventions. Furthermore, the need for hospitalization due to complications can lead to additional healthcare costs and lost productivity for patients, who may be unable to work during their recovery (2).

The prevalence of diabetes is escalating worldwide at a concerning pace, with the International Diabetes Federation reporting 537 million individuals diagnosed with the condition in 2021. This figure is anticipated to grow to 643 million by 2030 and reach 783 million by 2045 (3). In Africa, over fifty percent of adults with diabetes are found in some of the continent’s most populous countries, such as South Africa, Nigeria, and Ethiopia (4).

Foot complications are among the most severe and costly consequences of diabetes mellitus (DM), often leading to lower limb amputations due to foot ulcers. They typically arise from peripheral neuropathy, which reduces foot sensation, and peripheral vascular diseases that hinder blood circulation, increasing the risk of infections and gangrene (5).

In developing countries, the rates of foot ulcers and amputations are strikingly high. Contributing factors like poverty, poor sanitation and hygiene, and the common practices of walking barefoot often combine to worsen the impact of diabetic foot injuries (6). If a foot ulcer goes untreated and does not heal, there is a risk of infection, and 5% to 24% of these ulcers could lead to limb amputation within six to eighteen months after the initial evaluation (7).

In many African countries with limited resources, diabetes significantly strains healthcare systems. Research indicates Africa has the world’s second-highest prevalence of diabetic foot ulcers at 7.2% among diabetics (8). A study in Sri Lanka found that over fifty percent of participants understood foot self-care principles, their actual practices were lacking, and only about two-thirds regularly inspected their feet, the only principle consistently followed (9).

A diabetic foot ulcer is the most common complication of diabetes, characterized by lesions in deeper tissues. These ulcers are often associated with neurological impairments and peripheral vascular disease, manifesting as full-thickness wounds below the ankle in individuals with diabetes (7). To mitigate risks, implementing self-care practices for diabetic foot management at home is essential to reduce the likelihood of foot ulcers (10).

Diabetic foot self-care practices include regularly checking footwear, avoiding walking without shoes, not wearing tight socks, maintaining foot hygiene through washing and drying, properly trimming toenails, and using moisturizers to prevent dryness (11). By diligently adhering to these practices, patients can significantly lower their risk of developing gangrenous or ulcerated feet. This proactive approach involves a combination of regular foot inspections, maintaining proper hygiene, and ensuring appropriate footwear, managing underlying health conditions, such as diabetes or circulatory issues, plays a crucial role in foot health (12).

However, a significant obstacle in preventing complications associated with diabetic foot is the insufficient awareness and education concerning the symptoms, risks, and early identification of diabetic foot problems. Many individuals with diabetes may not fully understand the importance of regular foot care or may be unaware of the specific symptoms that can indicate the onset of foot complications, such as numbness, tingling, or changes in skin color and temperature. This lack of knowledge can lead to resulting in more severe complications that could have been prevented with timely intervention (13).

However, there is no representative data on diabetic foot-care practices in Africa. Therefore, this systematic review and meta-analysis aimed to determine the pooled estimate of overall foot self-care practice and associated factors among diabetic patients in Africa. The result of this research serves as a foundational resource for developing national and international strategies, protocols, and guidelines. Ultimately, it will have a significant impact on preventing the risks of DM.

## 2 Methods

### 2.1 Search strategy

This systemic review was conducted to assess the pooled prevalence and associated factors of diabetic foot-care practices among diabetic patients in Africa. We thoroughly reviewed existing systematic reviews to avoid redundancy and conducted a detailed search for published studies across several databases, including PubMed, Cochrane Library, Google Scholar, Web of Science, African Journals Online, and Science Direct. A set of predetermined search terms was employed to facilitate a thorough search strategy that encompassed all pertinent studies. All components within the records and Medical Subject Headings were utilized to enhance the search in the advanced PubMed search interface. The search strategy was formulated and refined for various databases, utilizing essential Boolean operators in conjunction with initial keywords (“diabetes mellitus” OR “diabetic foot-care practice” OR “prevalence of diabetes” AND “associated factors” AND “Africa”). The meta-analysis was conducted following the guidelines established by the Preferred Reporting Items for Systematic Reviews and Meta-Analysis (PRISMA) (14) (**Supplementary Table S1**). All searches were limited to papers written in English and the last search in all databases was performed on the 22^th^ July 2024.

### 2.2 Study selection and eligibility criteria

This research included studies involving diabetic patients and focused on the prevalence of foot-care practices and their associated factors. All studies were conducted in Africa and published in English. However, despite multiple attempts to contact the original authors via email, the current study did not include research publications that were not fully accessible. Since it was impossible to assess the articles’ content without the full texts, this omission was required. Moreover, research from which it was difficult to obtain the required data was not included in study. Furthermore, the systematic review and meta-analysis purposefully left out research from developed countries, editorial letters, reviews, communications, and systematic reviews.

### 2.3 Outcome measurement

The main outcome of interest was the prevalence of foot self-care practices. The secondary outcome involved the identification of factors associated with these foot-care practices among diabetic patients, which were assessed using odds ratios derived from binary outcomes reported in the primary studies included in the analysis. The key factors included in this review were education level (lower versus higher educational status), knowledge (poor versus good), and, residence (urban versus rural).

### 2.4 Quality assessment and data extraction

Reference management software aggregates search results from multiple databases and helps eliminate duplicate articles. The Joanna Briggs Institute’s meta-analysis tool was used for critical appraisal (15). Data extraction was performed by YAA and KAG using a standardized checklist in Microsoft Excel. For prevalence, the checklist included the author’s name, publication year, country, sub-region, and study design. For associated factors, data were organized into two-by-two tables to compute the log odds ratio for each factor. Discrepancies were resolved with a third author, BWA, to reach a consensus. YAA supervised the extraction process.

The assessment tool contains eight criteria: It was evaluated using the JBI critical appraisal checklist options of “Yes,” and “No.” The risks for biases were classified as low (total score, 5 to 8) and high (total score, 0 to 4). The study scored 50% or higher on all quality-assessed items, which were considered low-risk and included in this review. Disagreements that arose during the full-text quality assessment were resolved through evidence-based discussion

(**Supplementary Table S2**).

### 2.5 Statistical methods and analysis

Using Microsoft Excel 2010, the crucial data was collected and then transferred into Stata-14 for further examination. The I2 statistic, which shows the percentage of total variance due to heterogeneity rather than random variation, was calculated to evaluate the studies’ heterogeneity. Here, zero, twenty-five percent, fifty percent, and seventy-five percent represent no, low, moderate, and high degrees of heterogeneity, respectively.

There was significant heterogeneity among the studies, according to the test statistic (I2 = 98.4%, p < 0.000). Therefore, a random effects model was used to evaluate the overall impact of prevalence of foot-care practices. The DerSimonian-Laird weighting approach was used to generate prevalence rates and odds ratios at a 95% confidence interval. Furthermore, a subgroup analysis based on regional differences was carried out. A sensitivity analysis of the effect of a single study on the pooled prevalence from the meta-analysis was conducted. Using a subjective funnel plot and an objective Egger’s test with a 5% significance level, the publication bias was investigated. Using the odds ratio with a 95% confident interval and a significance level of 5% for the P-value, the association between foot-care practices and variables was estimated. Graphs, tables, text, and a forest plot were used to display the results.

## 3 Results

### 3.1 Search and study selection

**Figure 1:**
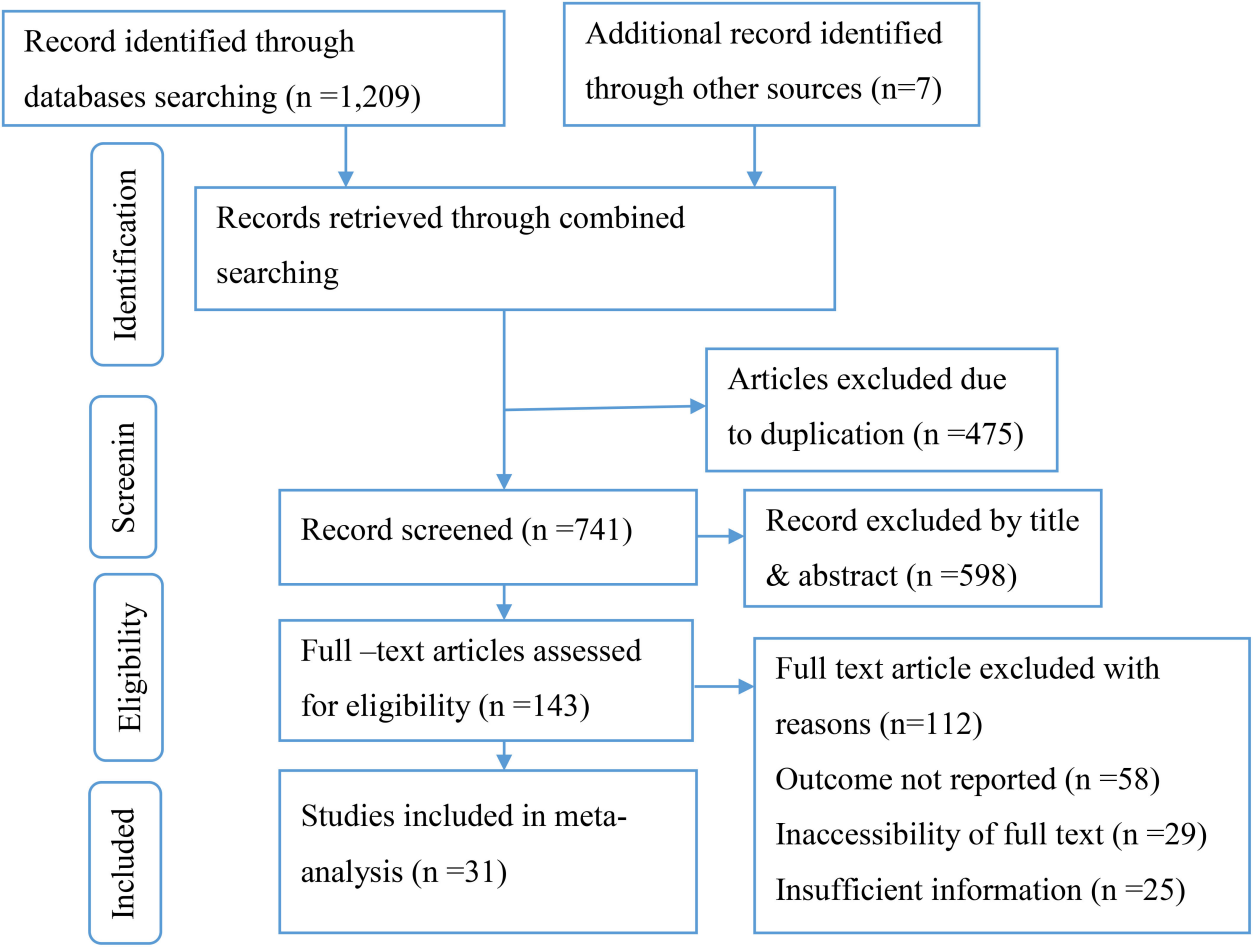
PRISMA flow diagram of included studies.

This review included published studies conducted on the prevalence of foot-care practices among diabetic patients in Africa. A total of 1,216 records were retrieved through electronic database searching. From these, 475 duplicated records were excluded, and from 741 articles screened using their titles and abstracts, 598 were excluded. 143, full-text articles were assessed for eligibility. From these, 112 full-text articles were excluded from *prior* criteria, and finally, 31 full-text primary articles were selected for quantitative analysis (**Figure 1**).

### 3.2 Characteristics of included studies

Thirty-one (16–46) African countries were represented in this review with a total of 8,759 diabetic patients. From all, eighteen of the studies were from eastern Africa (18–26, 31–34, 39–42, 46), eight were from western African countries (27–30, 35–38), three studies were from northern Africa (16, 17, 45), and two studies from Southern African countries (43, 44). The total sample size ranges from 68 to 513 (22, 24) (**Table 1**).

**Table 1:** Study characteristics of included studies.

S/N	Authors & Year	Country	Sub-region	Study-design	Sample-size	Prevalence of FCP (%)	QA
1	Elmansy et al. (2024) (16)	Egypt	North Africa	CS	84	42.9	LR
2	Abu-elenin et al. (2018) (17)	Egypt	North Africa	CS	264	37.8	LR
3	Negash et al. (2022) (18)	Ethiopia	East Africa	CS	267	53.9	LR
4	Betru et al. (2023) (19)	Ethiopia	East Africa	CS	420	66.7	LR
5	Seid et al. (2014) (20)	Ethiopia	East Africa	CS	313	56.2	LR
6	Hirpha et al. (2020) (21)	Ethiopia	East Africa	CS	370	82.7	LR
7	Tuha et al. (2021) (22)	Ethiopia	East Africa	CS	68	39	LR
8	Emire et al. (2022) (23)	Ethiopia	East Africa	CS	384	39.6	LR
9	Getie et al. (2020) (24)	Ethiopia	East Africa	CS	513	55.9	LR
10	Mekonen and Gebeyehu. (2020) (25)	Ethiopia	East Africa	CS	384	53.6	LR
11	Chali et al. (2018) (26)	Ethiopia	East Africa	CS	383	54.3	LR
12	Omotosho et al. (2024) (27)	Gambia	West Africa	CS	259	68.7	LR
13	Tuglo et al. (2021) (28)	Ghana	West Africa	CS	473	49	LR
14	Peprah et al. (2022) (29)	Ghana	West Africa	CS	220	47.7	LR
15	Afaya et al. (2022) (30)	Ghana	West Africa	CS	330	36.6	LR
16	Wamucii et al. (2020) (31)	Kenya	East Africa	CS	190	76.2	LR
17	Wanja et al. (2019) (32)	Kenya	East Africa	CS	133	48.8	LR
18	Mbisi et al. (2019) (33)	Kenya	East Africa	CS	301	45.1	LR
19	Nduati et al. (2022) (34)	Kenya	East Africa	CS	147	36.7	LR
20	Desalu et al. (2011) (35)	Nigeria	West Africa	CS	352	10.2	LR
21	Azeez and Emuze. (2022) (36)	Nigeria	West Africa	CS	100	23.1	LR
22	Magaji et al. (2024) (37)	Nigeria	West Africa	CS	241	17.8	LR
23	Okafor et al. (2024) (38)	Nigeria	West Africa	CS	382	51.3	LR
24	Chiwanga and Njelekela. (2015) (39)	Tanzania	East Africa	CS	404	15.6	LR
25	Nakidde et al. (2022) (40)	Uganda	East Africa	CS	228	56	LR
26	Kiruyi et al. (2023) (41)	Uganda	East Africa	CS	156	36.5	LR
27	Tusubira et al. (2023) (42)	Uganda	East Africa	CS	385	39.2	LR
28	Dikeukwu et al. (2013) (43)	South Africa	South Africa	CS	120	71.7	LR
29	Zwane et al. (2023) (44)	South Africa	South Africa	CS	402	23.5	LR
30	Adarmouch et al. (2020) (45)	Morocco	North Africa	CS	406	63.6	LR
31	Mukeshimana et al. (2015) (46)	Rwanda	East Africa	CS	80	54.9	LR

CS, Cross-Sectional; FCP, Foot Care Practice; QA, Quality Assessment; LR, Low Risk.

### 3.3 Pooled prevalence of diabetes foot self-care practice in Africa

**Figure 2:**
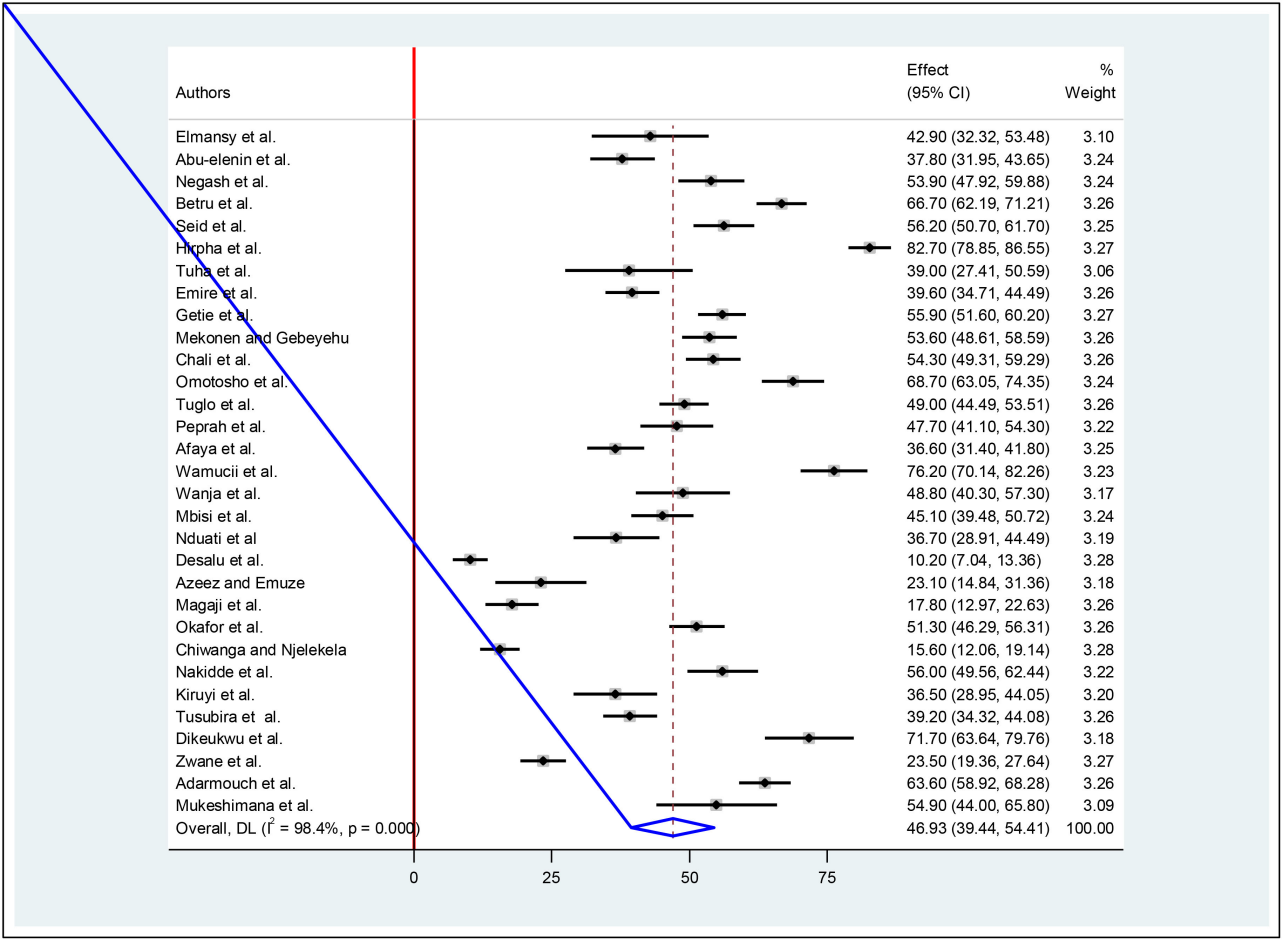
Forest plot of the pooled prevalence of foot self-care practices among diabetic patients in Africa.

The overall pooled prevalence of diabetes foot self-care practices among diabetic patients was 46.93% (95%CI (39.44–54.41)). This estimate was statistically significant with a P value of less than 0.001. Furthermore, heterogeneity between studies was found to be high with an I^2^ value of 98.4%, (**Figure 2**).

### 3.4 Sub-group meta-analysis

**Figure 3:**
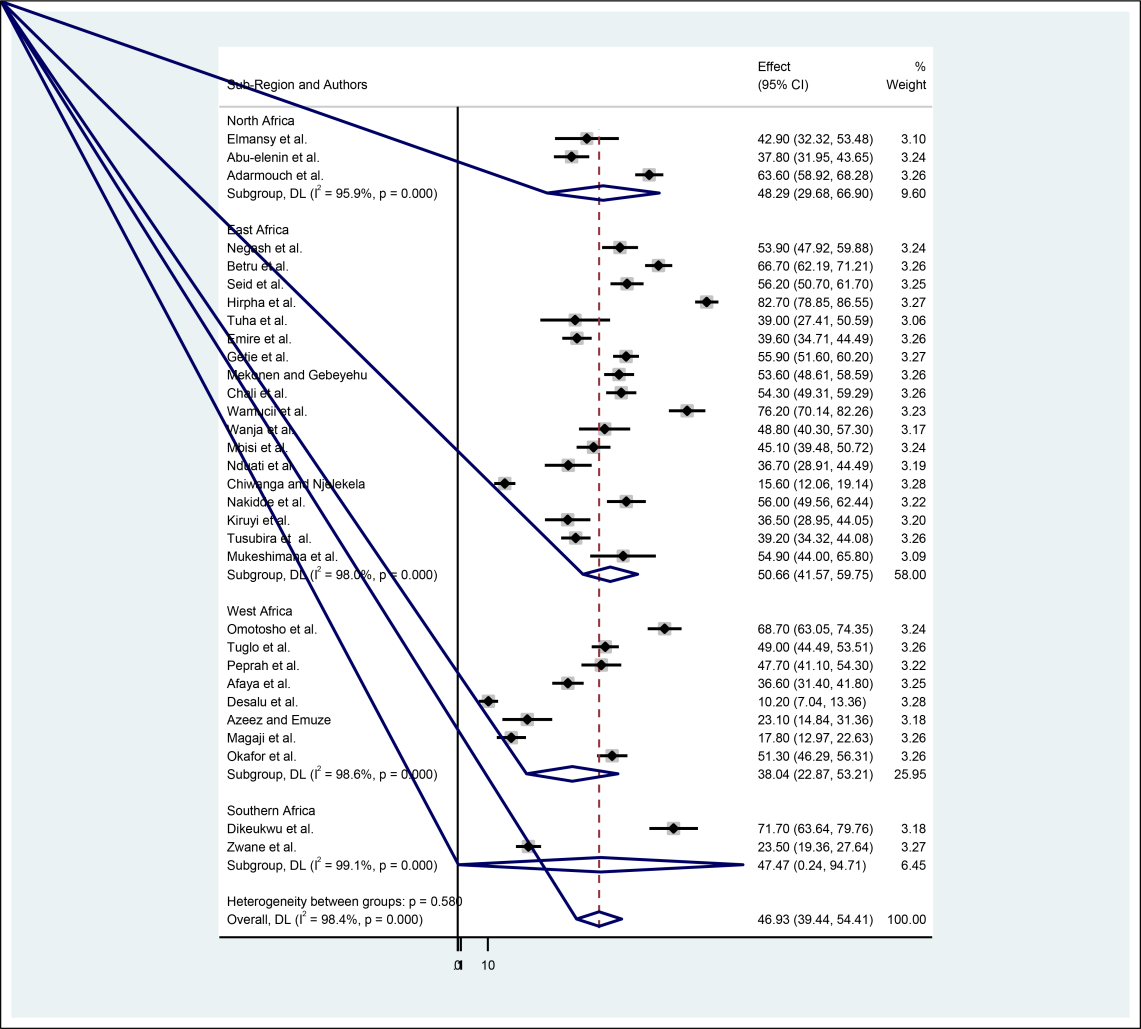
Sub-group level of diabetes foot self-care practices in Africa by sub-region.

To minimize random variations among the studies, we performed a sub-group analysis based on the regions where the studies were conducted. Based on sub-region, the highest pooled estimate of foot-care practices among diabetic patients was found in East Africa at 50.66% (95% CI: 41.57–59.75), while the lowest was in West Africa at 38.04% (95% CI: 22.87–53.21) (**Figure 3**).

### 3.5 Sensitivity meta-analysis

A sensitivity analysis using a random-effects model showed that no single study significantly influenced the overall meta-analysis results. The data presented in the table show that the estimates derived from an individual study align more closely with the aggregated estimate hence; there is no significant influence of any single study on the overall findings (**Table 2**).

**Table 2:** Sensitivity analysis of pooled prevalence to each study was removed one by one.

Study omitted	Estimate	95% Conf. interval	
Study of diabetes foot self-care practice			
Elmansy et al.	47.05	39.41	54.69
Abu-elenin et al.	47.23	39.52	54.93
Negash et al.	46.69	38.99	54.38
Betru et al.	46.25	38.67	53.84
Seid et al.	46.61	38.91	54.31
Hirpha et al.	45.70	38.75	52.66
Tuha et al.	47.17	39.54	54.80
Emire et al.	47.17	39.41	54.92
Getie et al.	46.62	38.87	54.37
Mekonen and Gebeyehu	46.70	38.96	54.43
Chali et al.	46.67	38.95	54.40
Omotosho et al.	46.19	38.62	53.76
Tuglo et al.	46.85	39.07	54.63
Peprah et al.	46.89	39.20	54.59
Afaya et al.	47.27	39.54	55.00
Wamucii et al.	45.94	38.46	53.43
Wanja et al.	46.86	39.20	54.52
Mbisi et al.	46.98	39.25	54.71
Nduati et al	47.26	39.59	54.92
Desalu et al.	48.17	41.35	55.00
Azeez and Emuze	47.70	40.09	55.31
Magaji et al.	47.90	40.39	55.41
Okafor et al.	46.77	39.03	54.52
Chiwanga and Njelekela	47.98	40.69	55.28
Nakidde et al.	46.62	38.94	54.29
Kiruyi et al.	47.26	39.60	54.93
Tusubira et al.	47.18	39.42	54.94
Dikeukwu et al.	46.11	38.53	53.68
Zwane et al.	47.71	40.11	55.32
Adarmouch et al.	46.36	38.72	54.00
Mukeshimana et al.	46.67	39.04	54.30
Combined	46.92	39.43	54.41

### 3.6 Meta-regression

A meta-regression analysis was conducted due to significant heterogeneity, with I-square values below 0.05. The aim was to identify sources of this heterogeneity for better result interpretation. However, the analysis found no significant variables related to sample size, publication year, or study sub-regions. Thus, the heterogeneity may stem from unaddressed factors (**Table 3**).

**Table 3:** Meta-regression analysis of factors affecting between-study heterogeneity.

Source of heterogeneity	Coefficient	Standard error	P-value
Year of Publication	0.83	0.06	0.63
Country	0.78	0.12	0.42
Sample size	0.89	0.04	0.51
Sub-region	1.04	0.43	0.92

### 3.7 Publication bias

**Figure 4:**
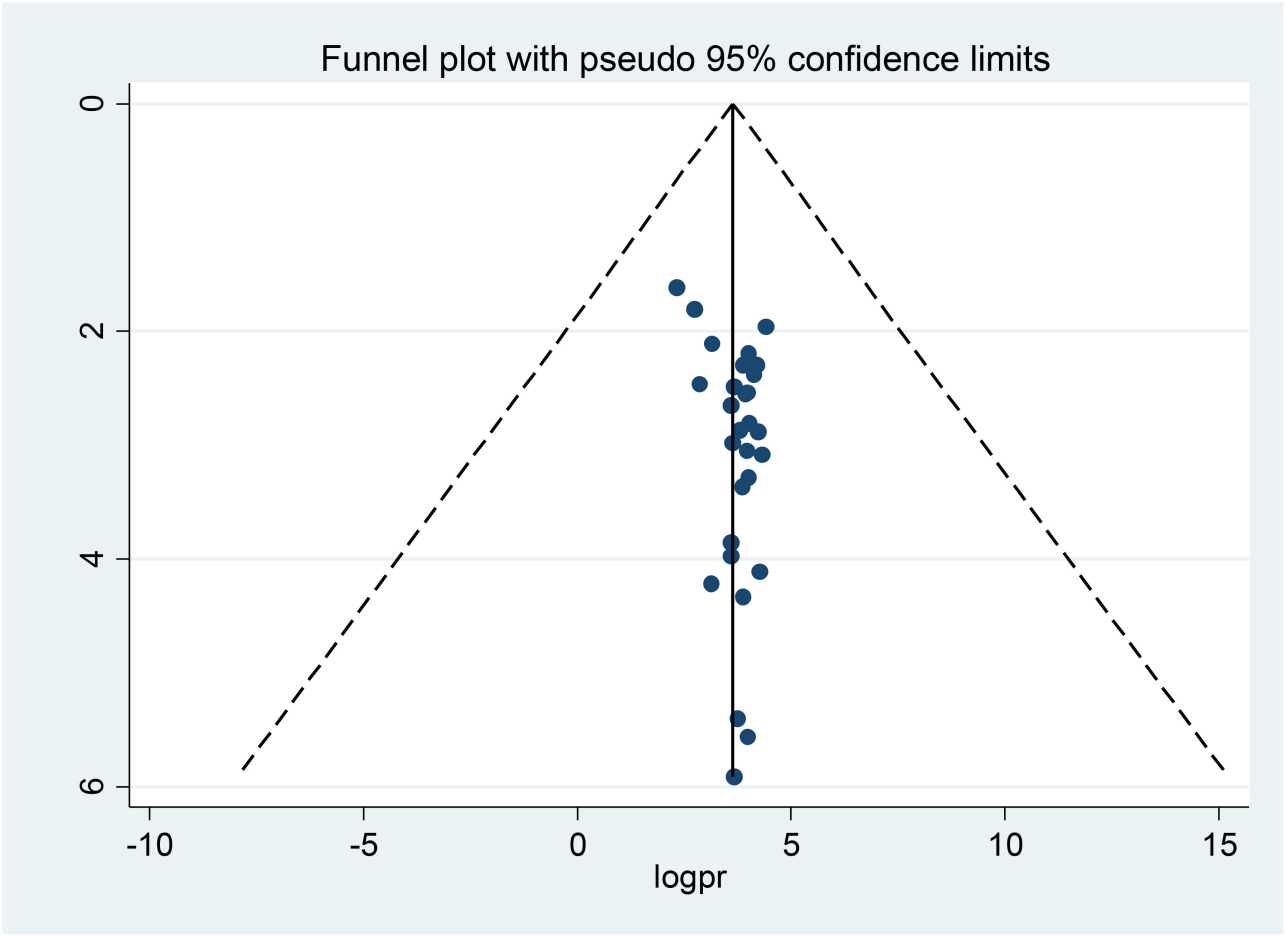
Funnel plots for publication bias of diabetes foot self-care practice in Africa, 2024.

The distribution of foot self-care practices among diabetic patients was examined for asymmetry through a visual inspection of the forest plot presented as a funnel plot. Furthermore, Egger’s and Begg’s regression test results demonstrated the non-existence of publication bias (p=0.53), and (P=0.34) respectively (**Figure 4**).

### 3.8 Factors associated with a diabetic foot self-care practice in Africa

A meta-analysis was conducted to determine the factors associated with foot self-care practices using a random effects model. Consequently, we analyzed the combined effect of three specific factors, knowledge, educational attainment, and place of residence on the outcome variable.

#### 3.8.1 Association between diabetic foot self-care practice and level of knowledge

To examine the association between diabetic foot-care practices and the knowledge of participants, a total of six studies were incorporated into the meta-analysis (20, 23–26, 44). The pooled results of the meta-analysis indicated a significant association between foot self-care practices and the knowledge of participants. Consequently, diabetic patients possessing a strong understanding of foot self-care practices were found to be 3.41 times more likely to be involved in these practices compared to poor knowledge (AOR: 3.41, 95% CI (2.22–5.23)). Moderate heterogeneity was observed across studies (I^2^ = 62.8%, P=0.002) for this reason, we used a random effects model.

#### 3.8.2 Association between diabetic foot self-care practice and educational level

A total of nine studies were incorporated into the meta-analysis to determine the association between diabetic foot-care practices and the level of education (17, 19, 22, 25–28, 44, 45). The studies included in the analysis indicated that individuals with a college education or higher were 3.00 times more likely to engage in diabetic foot self-care practices than those who were illiterate (AOR: 3.00, 95% CI (2.07–4.34)). Moderate heterogeneity was observed across studies (I^2^ = 50.4%, P = 0.004) for this reason, we used a random effects model.

#### 3.8.3 Association between diabetic foot self-care practice and residence

To identify the association between diabetic foot-care practice and residence, five studies were included in the meta-analysis (22, 23, 25, 41, 44). Accordingly, the pooled findings of the meta-analysis showed that living in rural was significantly associated with diabetic foot-care practices. As a result, diabetic patients who were living in rural residences were 2.50 times more likely to practices foot self-care as compared to diabetic patients who live in an urban area (AOR:2.50, 95% CI (1.65–3.80)). Higher heterogeneity was observed across studies (I^2^ = 93.2%, P=0.001), for this reason, we used a random effects model (**Table 4**).

**Table 4:** Factors associated with a diabetic foot self-care practice in Africa.

S.N.	Factors	Authors, Year and, *I^2^* with P-value	Odd Ratio (95%CI)
1	Level of knowledge	Said et al. (2014)	7.50 (3.83–14.71)
		Chali et al. (2018) (26)	5.01 (2.44–10.28)
		Emire et al. (2022) (23)	2.40 (1.31–4.40)
		Getie et al. (2020) (24)	2.14 (1.37–3.35)
		Mekonen and Gebeyehu. (2020) (25)	2.19 (1.07–4.47)
		Zewane et al. (2023)	4.49 (2.07–9.74)
		Overall, DL (*I^2^ =* 62.8%, P=.002)	3.41 (2.22–5.23)
2	Educational level	Abu-elenin et al. (2018) (17)	1.04 (1.29–3.68)
		Betru et al. (2023)	4.26 (2.53–7.18)
		Tuha et al. (2021) (22)	10.42 (2.50–43.47)
		Chali et al. (2018) (26)	3.63 (1.33–9.90)
		Tuglo et al. (2021) (28)	5.66 (1.89–16.94)
		Zewane et al. (2023)	3.86 (1.47–10.15)
		Adarmouch et al. (2020) (45)	1.81 (1.27–2.58)
		Mekonen and Gebeyehu. (2020) (25)	2.35 (1.01–5.45)
		Omotosho et al. (2024) (27)	2.65 (1.39–5.06)
		Overall, DL (*I^2^ =* 50.4%, P=.004)	3.00 (2.07–4.34)
3	Place of residences	Tuha et al. (2021) (22)	1.85 (1.10–3.09)
		Emire et al. (2022) (23)	7.16 (3.31–15.47)
		Mekonen and Gebeyehu. (2020) (25)	3.84 (1.91–7.73)
		Kiruyi et al. (2023) (41)	2.50 (1.19–5.25)
		Zewane et al. (2023)	2.97 (1.47–5.99)
		Overall, DL (*I^2^ =* 93.2%, P= .000)	2.50 (1.65–3.80)

## 4 Discussions

This systematic review and meta-analysis was conducted in response to the insufficient evidence regarding the prevalence of good foot-care practices. The primary objective was to estimate the pooled prevalence of foot-care practices and identify the associated factors among diabetic patients in Africa. Effective self-care is crucial for the management of diabetes mellitus, encompassing various components, including foot care (47). practice of self-care is not only important to diabetic patients but also for other medical practices to reduce the direct and indirect costs of medicine (48).

In this meta-analysis, the pooled estimate of overall foot self-care practice among diabetic patients in Africa was 46.93%. The finding was consistent with a previous study conducted in Malaysia (47.8%) (49). However, this finding was much lower than studies conducted in Thailand (87%), and Iran (74%) (50, 51), and higher than the study reported in Spain (30.2%), and Turkey (29.5%) (52, 53). Cultural disparities, economic variations, lifestyle differences, unequal healthcare access, and public education levels may explain the observed differences. Additionally, diverse methodologies for assessing outcomes across countries could contribute to these discrepancies. Furthermore, the varying educational levels and understanding of diabetes foot self-care among patients in different countries likely influence the differences in diabetes foot self-care practices.

This systematic review and meta-analysis also identified factors associated with diabetic foot care practices. Diabetic patients living in rural areas were nearly three times more likely to have poor foot self-care practices than those who live in urban areas. This finding is supported by a study conducted in India (54). The possible reason for those diabetic patients in rural areas often have limited access to health education resources, leading to lower awareness of self-care practices compared to urban patients. In Africa, many rural diabetics are farmers who walk barefoot, increasing their risk of bites and injuries that can lead to foot ulcers. The lack of accessible self-care information further reduces their proactivity in addressing foot issues, heightening the risk of developing foot problems.

In addition, the current research indicates that diabetic patients with limited literacy skills and primary education were three times more likely to exhibit inadequate foot self-care practices compared to their counterparts who had attained college education or higher. Comparable results have been documented in studies carried out in Vietnam and Indonesia (55, 56). As a diabetic patient’s level of education increases, likely, their understanding of foot self-care principles will also enhance. This understanding is crucial for the prevention of diabetes mellitus, which is best managed through consistent practices of foot self-care. Furthermore, individuals with higher educational attainment are generally more inclined to seek out and comprehend information related to their condition, foot care, as well as to recognize the guidance provided by their healthcare providers.

In this study, participants who had good knowledge of foot-care practices were three times more likely to practice foot-care as compared to poorly knowledgeable. The findings of this study are further supported by a similar investigation conducted in India, which also demonstrated an association between knowledge of foot-care practices and the likelihood of engaging in such activities (54). The potential rationale for this is that effective diabetes self-care practices require the active participation of both individuals living with diabetes and healthcare professionals. This collaboration aims to enhance best practices by improving knowledge and comprehension of the condition, as well as anticipating the long-term consequences associated with the disease.

A comprehensive search strategy was used for this systematic review and meta-analysis, including both published and unpublished studies. A random-effects model was used to address the potential variability across studies. However, as a limitation, the results of this study should be interpreted by considering possible limitations, such as the restriction of studies written in English limited the number of studies included in a meta-analysis and the limited number of studies conducted in Africa may hinder the ability to generalize findings to the entire population. This review exhibited significant interestedly heterogeneity, which could not be addressed through subgroup analysis. Additionally, all included studies were cross-sectional, which may have introduced confounding factors that weaken the causal inferences about the relationship between foot self-care practices and associated factors.

## 5 Conclusions

In conclusion, more than half of diabetic patients had poor diabetes foot self-care practices. Due to having low educational status, living in a rural area, and a level of knowledge among diabetic patients. It is better to give health education to diabetic patients and their caregivers about the basic principles of diabetes foot care, like regular inspection of the feet and appropriate footwear, as well as how to encourage diabetic patients to adhere to foot self-care practices at their regular follow-up time.

## Data Availability

The original contributions presented in the study are included in the article/**Supplementary Material**. Further inquiries can be directed to the corresponding author.
