# Supplementary material for: Prevalence and associated factors of foot self-care practice among diabetic patients in Africa: systematic review and meta-analysis
Source: Front Endocrinol (Lausanne). 2025 Jun 4;16:1527402. doi: 10.3389/fendo.2025.1527402 (PMC12173898; doi:10.3389/fendo.2025.1527402)
Supplement: Supplementary file 2 [file Table2.docx]

**S2-Table**. Quality assessment for the included Studies

| **Item** | **Clearly defined incion criteria** | **Describe the study setting and participant** | **Valid and reliable exposure measurement** | **Objective and standard criteria for measurement** | **Identified confounder** | **Strategies to deal with confounders** | **Valid and reliable outcome measurement** | **Appropriate static analysis** | **No of ‘yes’ ‘** |
| --- | --- | --- | --- | --- | --- | --- | --- | --- | --- |
| Elmansy et al. | Yes | Yes | No | Yes | Yes | No | Yes | Yes | 6/8=75 |
| Abu-elenin et al. | Yes | Yes | Yes | Yes | No | No | Yes | Yes | 6/8=75 |
| Negash et al. | Yes | Yes | No | Yes | Yes | No | Yes | Yes | 6/8=75 |
| Betru et al. | Yes | Yes | No | Yes | Yes | Yes | Yes | Yes | 7/8=87.5 |
| Seid et al. | Yes | Yes | No | Yes | Yes | Yes | Yes | Yes | 7/8=87.5 |
| Hirpha et al. | Yes | Yes | Yes | Yes | Yes | No | Yes | Yes | 7/8=87.5 |
| Tuha et al. | Yes | Yes | No | Yes | Yes | Yes | Yes | Yes | 7/8=87.5 |
| Emire et al. | Yes | Yes | Yes | Yes | No | No | Yes | Yes | 6/8=75 |
| Getie et al. | Yes | Yes | No | Yes | Yes | Yes | Yes | No | 6/8=75 |
| Mekonen and Gebeyehu | Yes | Yes | Yes | Yes | No | Yes | Yes | No | 6/8=75 |
| Chali et al. | Yes | Yes | Yes | Yes | No | No | Yes | Yes | 6/8=75 |
| Omotosho et al. | Yes | yes | No | Yes | Yes | Yes | Yes | Yes | 7/8=87.5 |
| Tuglo et al. | Yes | Yes | Yes | No | Yes | No | Yes | Yes | 6/8=75 |
| Peprah et al. | Yes | Yes | Yes | Yes | Yes | Yes | Yes | No | 7/8=87.5 |
| Afaya et al. | Yes | Yes | Yes | Yes | Yes | Yes | No | Yes | 7/8=87.5 |
| Wamucii et al. | Yes | Yes | No | Yes | Yes | Yes | Yes | Yes | 7/8=87.5 |
| Wanja et al. | Yes | Yes | No | Yes | No | Yes | Yes | Yes | 6/8=75 |
| Mbisi et al. | Yes | Yes | Yes | Yes | Yes | Yes | Yes | No | 7 /8=87.5 |
| Nduati et al | Yes | Yes | No | Yes | Yes | No | Yes | Yes | 6/8=75 |
| Azeez and Emuze. | Yes | Yes | Yes | Yes | Yes | Yes | Yes | No | 7/8=87.5 |
| Magaji et al. | Yes | Yes | Yes | No | Yes | Yes | Yes | No | 6 / 8=75 |
| Okafor et al. | Yes | Yes | No | Yes | Yes | Yes | Yes | Yes | 7/8=87.5 |
| Chiwanga and Njelekela. | Yes | Yes | No | Yes | Yes | Yes | Yes | Yes | 7/8=87.5 |
| Nakidde et al. | Yes | Yes | No | Yes | Yes | Yes | Yes | Yes | 7/8=87.5 |
| Kiruyi et al. | Yes | Yes | Yes | Yes | Yes | Yes | Yes | No | 7/8=87.5 |
| Tusubira et al. | Yes | Yes | Yes | No | Yes | Yes | No | Yes | 6/8=75 |
| Dikeukwu et al. | Yes | Yes | Yes | No | Yes | Yes | Yes | Yes | 7/8=87.5 |
| Zwane et al. | Yes | Yes | No | Yes | Yes | Yes | Yes | No | 6/8=75 |
| Adarmouch et al. | Yes | Yes | Yes | Yes | Yes | Yes | Yes | No | 7/8=75.5 |
| Mukeshimana et al. | Yes | Yes | No | Yes | Yes | Yes | No | Yes | 7/8=75.5 |
